# A Comparative Study of BDS Triple-Frequency Ambiguity Fixing Approaches for RTK Positioning

**DOI:** 10.3390/s21072565

**Published:** 2021-04-06

**Authors:** Huizhong Zhu, Yangyang Lu, Longjiang Tang, Jun Li, Aigong Xu, Maorong Ge

**Affiliations:** 1 School of Geomatics, Liaoning Technical University (LNTU), Fuxin 123000, China; 471820580@stu.lntu.edu.cn (Y.L.); 471710030@stu.lntu.edu.cn (L.T.); 472010048@stu.lntu.edu.cn (J.L.); xuaigong@lntu.edu.cn (A.X.); 2 German Research Centre for Geosciences (GFZ), 14473 Potsdam, Germany; maor@gfz-potsdam.de

**Keywords:** BDS, long range RTK, triple-frequency observations, carrier phase ambiguity, LAMBDA, integer ambiguity resolution

## Abstract

Concerning the triple-frequency ambiguity resolution, in principle there are three different realizations. The first one is to fix all the ambiguities of the original frequencies together. However, it is also believed that fixing the combined integer ambiguities with longer wavelength, such as extra-wide-lane (EWL), wide-lane (WL), should be advantageous. Also, it is demonstrated that fixing sequentially EWL, WL and one type of original ambiguities provides better results, as the previously fixed ambiguities increase parameters’ precision for later fixings. In this paper, we undertake a comparative study of the three fixing approaches by means of experimental validation. In order to realize the three fixing approaches from the same information in terms of adjustment, we developed a processing strategy to provide fully consistent normal equations. We first generate the normal equation with the original undifferentiated carrier phase ambiguities, then map it into that with the combined and double-differenced ambiguities required by the individual approach for fixing. Four baselines of 258 m, 22 km, 47 km and 53 km are selected and processed in both static and kinematic mode using the three ambiguity-fixing approaches. Indicators including time of first fixed solution (TFFS), the correct fixing rate, positioning accuracy and RATIO are used to evaluate and investigate results. We also made a preliminary theoretical explanation of the results by looking into the decorrelation procedure of the ambiguity searching algorithm and the intermediate results. As conclusions, integrated searching of original ambiguities or combined ambiguities has almost the same fixing performance, whereas the sequential fixing of EWL, WL and B1 ambiguities overperforms the integrated searching. By the way, the third-frequency data can shorten the TFFS significantly but can hardly improve the positioning.

## 1. Introduction

Along with the development of real-time kinematic (RTK) positioning, the distance between users and the reference station is always a challenge in order to enlarge its service coverage. The crucial issue is the Integer Ambiguity Resolution (IAR) which is the key for fast and precise global navigation satellite system (GNSS) positioning. The phase observations with fixed ambiguity is equal to ultra-precise range observations and improve the other parameters remarkably, so that positioning accuracy of centimeter- or even millimeter-level can be achieved [1]. This challenge still remains a research hotspot in network RTK or PPP-RTK, since on the one hand, it could be applied to the ambiguity resolution of reference networks, and on the other hand, there are still a lot of regions with only very sparse reference stations.

In principle IAR performance depends heavily on the observation noise and modelling errors, wavelength of the ambiguity parameters and the observing geometry. The smaller the observation noise or the larger the wavelength, the better is the fixing. A stronger observing geometry will provide a better float solution, and consequently a more reliable fixing. However, several factors which cannot be efficiently removed by between-station differencing will remain in the observations used in the adjustment. The most significant ones are the tropospheric and ionospheric delays which are increasing with the inter-station distance [2]. They can be corrected with a priori correction models [3,4], respectively, however these corrections are not accurate enough for precise positioning, especially, the feasibility of ambiguity resolution in precise positioning is still related to the state of the ionosphere and can be affected by ionospheric disturbances of different scales [5]. Thus, additional parameters must be introduced and estimated simultaneously with the other parameters. This degrades the strength of the float solution and makes the instantaneous ambiguity resolution very difficult.

Thereafter, on the one hand, a number of studies worked on providing possible accurate ionospheric and tropospheric delay information and imposing proper constraints on them to retain the strength of the solution and reduced observation residuals and demonstrated their efficiency via the resulting ambiguity fixing performance [6,7,8]. On the other hand, from the processing of short baselines where dual-frequency data can be applied as independent observations, due to the very strong constraint among ambiguities of different frequencies, ambiguities can be fixed very quickly and reliably [9]. Fortunately, the third-frequency observations available in the modernized GPS and the newly emerging system BDS and Galileo bring obviously additional information on ionospheric delays and will certainly enhance the constraint among ambiguities at different frequencies [10,11,12]. The ambiguity resolution of multi-frequency are investigated thoroughly together with the consideration of multi-constellations, as both sides rely tightly on each other through more information in ionospheric delays.

Beside strengthening the solution and reducing the errors to increase the fixing opportunity as discussed above, to quickly and precisely find out the correct integer solution is of the same importance, i.e., the fixing approaches. In the past, several approaches were developed, most of them are based on searching the optimal integer ambiguities statistically closest to the corresponding float solution [9,10,11,12,13,14,15,16,17]. The mostly used approach is the Least-Squares AMBiguity Decorrelation Adjustment (LAMBDA) method [1], for example, which is also applied in the recent contributions [18,19,20].

A large number of scholars have studied the long-range RTK positioning using triple-frequency observations with the concentration of triple-frequency ambiguity resolution and observation modelling as well, One group of scientists tried to resolve the ambiguities directly based on a set of optimized combinations of the phase and range observations, which is referred as triple-frequency carrier-phase ambiguity resolution (TCAR). It is proved that EWL and WL can be fixed reliably, however the last one with a rather shorter wavelength can only be fixed using geometrical observations and ambiguity searching approaches. It is also confirmed TCAR or the later improved method does not have obvious advantage to some of the searching schemes [21]. For example, [12,22] has studied the contribution of the third-frequency of GPS and Galileo on long-range RTK ambiguity resolution, the results of a cascading fixing scheme in the sequence of EWL, WL and L1/E5 ambiguities are very promising although only simulated observations were available and employed.

Overall, there are also several different schemes for multi-frequency ambiguity resolution: (1). to searching all ambiguities of the original frequencies, and (2) to map the ambiguities to extra-wide lane (EWL), wide-lane (WL) and one original frequency and to search the integer solution altogether, (3) similar to (2) but search the ambiguities of the same wavelength in batch with that of the longest wavelength first. Usually the second one is believed better than the first one as the EWL and WL ambiguities should be easily fixed due to their longer wavelength. However, there is also the opinion that LAMBDA should give the same result as the first two schemes are based on the same float solution information. A comparative study of the three schemes is the major goal of this contribution.

The Chinese BeiDou Navigation Satellite System (BDS) should be the first one of the GNSS which provides triple-frequency data across the constellation. In fact, it can provide signals at more than triple frequencies since 27 December 2018 [23]. Nowadays, Galileo and all GPS Block IIF and III satellites are also transmitting signals at third or even more frequencies. In this contribution, the comparative study on the above-mentioned three fixing schemes is carried out based on the BDS triple-frequency data. In order to do a fair comparison, all the fixing will start from exactly the same normal equation information. We use double-differenced but uncombined observations with undifferenced ambiguities at the original frequencies, which should be equivalent to that using undifferenced or single-differenced observations if their covariance matrix is rigorously considered [24]. Before the ambiguity resolutions these ambiguities are mapped to the combination among frequencies, i.e., EWL, WL etc. and between stations and satellites, i.e., double-differenced ambiguities for fixing. The time of first fixed solution (TFFS) and the correct fixing rate, and of course also the derived positioning deviations are employed as fixing performance indicators for evaluation. The result of the comparison will be further investigated by examining the theory and algorithm of the ambiguity resolution schemes. The paper is arranged as follows: the related functional and stochastically models will be introduced with the handling of atmosphere delays after this introduction. Then, ambiguity mapping method is presented for both among frequencies and between stations and satellites. Three ambiguity fixing schemes are defined for comparison based on the ambiguity searching approach. Afterwards, data processing strategy is presented, and results are compared and analyzed before conclusions are drawn.

## 2. Estimating Model

For a better understanding of the ambiguity resolution schemes, we briefly introduce the observation equations, and the handling of tropospheric and ionospheric delays, as well as ambiguity mapping for triple-frequency data processing.

### 2.1. Observation Equation

The observation equation of GNSS multi-frequency undifferenced and uncombined carrier phase can be expressed as:

(1) $$\phi_{i,r}^{p}-\rho_{r}^{p}=H_{r}^{p}\cdot X_{r}+c\cdot (dt_{r}-dt^{p})+M_{r}^{p}\cdot T_{r}-\mu_{i}\cdot I_{1,r}^{p}+\lambda_{i}^{}\cdot B_{i,r}^{p}+\varepsilon_{i,r}^{p},P_{i,r}^{p}$$

where p, r and i are the indicates of the satellite, receiver and carrier phase frequency, respectively; $X_{r}$ is the coordinate parameters vector after first order Taylor expression with design matrix $H_{r}^{p}$; $\phi_{i,r}^{p}$ is the carrier phase observation, $\rho_{r}^{p}$ is the geometric distance from the satellite to the receiver r; $dt_{r}$ and $dt^{p}$ are the clock error of the receiver and satellite, respectively; c represents the speed of light; $T_{r}^{}$ is the zenith tropospheric delay; $M_{r}^{p}$ is tropospheric mapping function; $\mu_{i}$ is the ionospheric coefficient for frequency i, $\mu_{i}=f_{1}^{2}/f_{i}^{2}$; $I_{1,r}^{p}$ is the ionospheric delay of the first frequency, and $\lambda_{i}^{}$ is the wavelength of carrier phase; $B_{i,r}^{p}$ is the carrier phase ambiguity, $\varepsilon_{i,r}^{p}$ is the measurement noise, $P_{i,r}^{p}$ is the weight matrix of the observations and which value is calculated according to the altitude angle of the satellite.

In the baseline or RTK processing, the single-differenced (SD) observation between stations can be applied, as it can eliminate the satellite clock biases and reduce the effect of satellite orbit biases and atmospheric delays. In order to also eliminate the receiver clock bias, further difference between satellites is usually employed in practice, i.e., double-differenced (DD) observations are used. Suppose that two stations simultaneously observe the reference satellite q, and the station coordinate and zenith tropospheric delay at the reference stations are not estimated, only those at the moving station are estimated, the DD observation equations read as:

(2) $$v_{i,rs}^{pq}=H_{r}^{pq}\cdot X_{r}+M_{r}^{pq}\cdot T_{r}-\mu_{i}\cdot I_{1,rs}^{p}+\mu_{i}\cdot I_{1,rs}^{q}+\lambda_{i}\cdot B_{i,rs}^{p}-\lambda_{i}\cdot B_{i,rs}^{q}-(\phi_{i,rs}^{pq}-\rho_{i,rs}^{pq})$$

where r,s represent rover station and reference station, respectively, $v_{i,rs}^{pq}$ represents observation residual. As is well known, using DD or SD or undifferenced observations are equivalent if their covariance matrices are considered rigorously [24].

Usually, DD ambiguities should be involved in the DD observation equations, as they have the nature integer characteristics and can be fixed directly. However, the DD ambiguities may change along with satellite rises and sets, so the normal equation must be reformed accordingly. This makes the realization of the algorithm much more inconvenient. Therefore, SD ambiguity parameters are used in this study and then are mapped into DD ambiguities before integer ambiguity resolution.

### 2.2. Atmospheric Delay Estimation

The atmospheric delay is one of the major error sources that limits the accuracy and reliability of RTK positioning and the impact becomes more serious as the inter-station distance increases, since both troposphere and ionosphere delays can be neither cancelled by between stations differencing nor precisely corrected. For tropospheric delays large height differences between stations can also cause similar inaccurate modelling due to the different path of the transmitting signals [25].

In practice, the dry component of the tropospheric delay can be corrected by a model, for example the Saastamoinen model using pressure and temperature from GPT2 [26,27], whereas the wet component is parameterized as either piece-wise liner function or random-walk process to be estimated. Regarding to the mapping functions, the mostly used are GMF and VMF1 and the later one is used in this study [28,29].

Ionosphere delays can be eliminated by forming the ionosphere-free combination from observations of at least two frequencies. This is very often applied in long-range baseline data processing and the ambiguity of ionosphere-free observations must be separated into wide-lane and narrow-lane and fixed sequentially [30,31]. A more advantageous strategy is to use uncombined observations with slant ionospheric delays as unknown where the ambiguity of original frequencies can be estimated by imposing constraints on spatial and/or temporal ionospheric delay variations [32]. The later one is extremely suitable for triple-frequency data processing to avoid the selection of ionosphere-free combinations to involved in the data processing. Therefore, the later one is employed in this study with temporal constraint, i.e., inter-epoch ionospheric delay parameter constraint.

The random walk process for both ionospheric and tropospheric delays can be mathematically expressed as:

(3) $$\begin{array}{l} d(t_{k+1})=d(t_{k})+w(t_{k})\\ E(d(t_{0}))=0,Cov(d(t_{0}))=\sigma_{}^{2}\\ E(w(t_{k}))=0,Cov(w(t_{k}))=q^{2}(k)\cdot (t_{k}-t_{k-1}) \end{array}$$

where E(⋅), Cov(⋅) are the functions for expectation and, variance, respectively, and t is epoch time. The symbols $d,w,\sigma^{2}$, and q denote residual value, change of residual value, variance and power density of the corresponding atmospheric parameter, respectively. For RTK positioning, the remaining delay in the SD observations between stations depends on the inter-station distance, so the power density should be fine-tuned according to the baseline length. For example, both ionospheric and zenith tropospheric delays are almost fully eliminated for short baselines, the constraint of the initial state and the power density should be very close to zero, so that all estimates of state parameter are zero. In practice, the power density of the random walk processes can be sophistically adjusted in order to optimize the positioning performance, for example, fine-tuning the power density during the initial period to shorten the ambiguity convergence time and to improve positioning accuracy and reliability [33]. It can also be adapted according to the ionospheric variation calculated from ionosphere combinations of phase observations or the estimated delay parameters in the previous epochs [34].

### 2.3. Parameter Estimation

The DD observation equation system of Equation (3) is abbreviated for the presentation of parameter estimation and ambiguity resolution as:

(4) v=Ax+Dy+Cb+l, P

where *x* represent position parameters; y includes the residual zenith tropospheric delay after applying corrections from VMF1 model and the slant ionospheric delay parameters each for one satellite, i.e., ${\left(T_{r},I_{1}^{1},I_{1}^{2},\cdots I_{1}^{nsat}\right)}^{T}$ with *nsat* as the number of satellites observed at the epoch, and will be estimated as random walk process as described in Section 2.2; and *b* represents SD ambiguity parameters; and A,D,C are the corresponding coefficient matrices for parameters *x*, *y* and *b*, respectively; l is pre-fit observation residuals. It should be mentioned that for kinematic positioning the position parameters are time-varying instead of constant parameters all over the time for the static positioning.

According to the least square’s criterion, the normal equation of Equation (5) is expressed as:

(5) $$\left[\begin{array}{ccc} A^{T}PA & A^{T}PD & A^{T}PC\\ & D^{T}PD & D^{T}PC\\ sym & & C^{T}PC \end{array}\right]\left[\begin{array}{c} x\\ y\\ b \end{array}\right]=\left[\begin{array}{c} A^{T}Pl\\ D^{T}Pl\\ C^{T}Pl \end{array}\right]$$

which is abbreviated as:

(6) $$\left[\begin{array}{ccc} N_{xx} & N_{xy} & N_{xb}\\ & N_{yy} & N_{yb}\\ sym & & N_{bb} \end{array}\right]\left[\begin{array}{c} x\\ y\\ b \end{array}\right]=\left[\begin{array}{c} W_{x}\\ W_{y}\\ W_{b} \end{array}\right]$$

This normal equation system is estimable even if a loose constraint on the reference ambiguity parameters is applied.

### 2.4. Ambiguity Mapping

As is mentioned, the ambiguities in Equations (5)–(7) are SD-ambiguities between the two stations at the original signal frequencies in order to avoid the complicated reformation due to satellites rises or sets. Since only DD-ambiguities have integer characteristics because of the existence of uncalibrated phase delays, the SD ambiguities must be mapped into DD ambiguities for integer ambiguity resolution. This re-parameterization can be realized by the normal equation mapping based on a defined parameter transformation proposed by Blewitt and Dong and Bock [30,31].

From a given set of SD ambiguities, a number of sets of independent DD-ambiguities can be defined. There are various criteria to select the ones to be fixed the most easily, for example according to their elevations and length of continuous observations etc. Obviously from SD to DD the mapping is rank-defect, independent SD ambiguities must be further added to complete the transformation to full-rank. Assume that a full rank transformation from SD b to DD $\hat{b}$ is:

(7) $$\hat{b}=Tb$$

then the corresponding normal equation system can be derived from Equation (6) as:

(8) $$\left[\begin{array}{ccc} N_{xx} & N_{xy} & N_{xb}\cdot T^{-1}\\ & N_{yy} & N_{yb}\cdot T^{-1}\\ sym & & T^{-T}\cdot N_{bb}\cdot T^{-1} \end{array}\right]\left[\begin{array}{c} x\\ y\\ \hat{b} \end{array}\right]=\left[\begin{array}{c} W_{x}\\ W_{y}\\ T^{-T}\cdot W_{b} \end{array}\right]$$

Besides the mapping from SD to DD ambiguities, ambiguities combination of various frequencies can also be implemented with this mapping approach. For the triple-frequency data processing, the mapping can be generalized defined as:

(9) $$T\cdot b=\left[\begin{array}{ccc} i_{1}\cdot I_{n} & j_{1}\cdot I_{n} & k_{1}\cdot I_{n}\\ i_{2}\cdot I_{n} & j_{2}\cdot I_{n} & k_{2}\cdot I_{n}\\ i_{3}\cdot I_{n} & j_{3}\cdot I_{n} & k_{3}\cdot I_{n} \end{array}\right]\cdot \left[\begin{array}{c} b_{1}\\ b_{2}\\ b_{3} \end{array}\right]=\left[\begin{array}{c} b_{\left(i_{1},j_{1},k_{1}\right)}\\ b_{\left(i_{2},j_{2},k_{2}\right)}\\ b_{\left(i_{3},j_{3},k_{3}\right)} \end{array}\right]=b^{\prime }$$

where $\left(i_{1},j_{1},k_{1}\right)$, $\left(i_{2},j_{2},k_{2}\right)$ and $\left(i_{3},j_{3},k_{3}\right)$ are the combination coefficients and they must be integer values, so that the mapped ambiguities also have integer characteristics for fixing. For example, with (0, 1, −1), (1, −1, 0), and (1, 0, 0). we obtain the EWL, WL and B1 as mapped ambiguities. The normal equation system with the mapped ambiguities can be derived in the same way as for the mapping from SD to DD ambiguities.

Usually, it is believed that ambiguities with longer wavelength can be fixed more easily. It might be true for most of the searching approaches but not for the integrated searching with LAMBDA method. This will be demonstrated and investigated in this study.

In principle, the former mapping, from SD to DD ambiguities, is definitely needed for integer ambiguity resolution, whereas the later one is carried out on demand.

## 3. Ambiguity Resolution

There are two widely used ambiguity fixing methods: the rounding method and the searching method. The rounding method, as is named, directly rounds the estimated float ambiguity to its nearest integer. This is the simplest method to obtain the integer ambiguity, however it requires the float estimate with a certain accuracy so that the rounding is reliable. Therefore, it is mostly used in static post processing with long-term observations. The searching method is to find the integer ambiguities mostly compatible to the float solution according to the float estimates and their covariance. The most famous one is the LAMBDA method proposed by Teunissen [1].

### 3.1. Ambiguity Searching Approaches

Statistically speaking, the float estimates and their covariance matrix of the ambiguities define the confidence ellipsoid to which the integer solution belongs. However, how to find out the integer candidate vectors from this confidence ellipsoid has always been a key issue, especially in the case that the quantity of the observations is rather small, and the observation geometry is very poor.

Before LAMBDA the searching methods define the search areas according to the STD of each individual ambiguity [1]. Due to high correlation of ambiguities, the searching area defined this way can be extremely large than the confidence ellipsoid, so that thousands of candidates are included and must be checked and results into very long computation time. Although it was improved by introducing further constraint on ambiguity differences [9], but it is still not good enough for ambiguity fixing using observations of a single epoch or few epochs [35]. The key aspect of LAMBDA method is to find out the best integer linear combination of original ambiguities, so that the correlation among them is at the lowest. This means the searching region can be rather precisely defined by the STD of each individual ambiguity, and only few candidates are identified and checked for fixing.

The LAMBDA method is widely used for integer ambiguity searching, especially for fast ambiguity fixing with short-term observations. It can make full use of the original observation information, as is proved, it can maximize the success rate of ambiguity fixing [14]. We use LAMBDA to search the best ambiguity in the study.

Instead of directly searching integer ambiguity, Gaussian integer transformation is carried out on the ambiguity matrix to reduce the correlation of the ambiguities in the LAMBDA method. Afterwards, the integer ambiguities are converted to the original to obtain the parameters of the fixed solution. Since the task of the LAMBDA approach is to find out the best integer combination among the original ambiguities, therefore it should reach the same integer combination in principle no matter the searching starts with ambiguities of the original frequencies or that containing EWL and WL in spite of that the later ones are usually believed to be fixed more easily. More specifically, transforming original ambiguities into them before applying LAMBDA is not necessary which will be investigated and validated later on.

### 3.2. Ambiguity Resolution Strategy

For the ambiguity resolution of a special positioning solution, the searching approach can be applied in two different ways: the first one is to search all ambiguities simultaneously, whereas the second is to do the searching in sequential batch, also referred as to stepwise approach or cascading approach. In the second approach, the set of ambiguities which can be fixed most likely is selected and fixed, and then the solution is updated with the fixed ambiguities. Obviously, the remaining ambiguities in the updated solution will have a better quality, so that they can be more easily and reliably fixed now. This procedure can be repeated until no more ambiguities can be fixed. Usually, taking the advantage of the long wavelength of EWL and WL ambiguities, they should be fixed firstly to improve the solution and consequently to assist in resolving the short-wavelength ambiguities. In fact, the partial fixing can be considered as a special case of this approach, in which difficultly to be fixed ambiguities, for example, ambiguities of a rising satellites with very few observations or at a very low elevation, or those with the largest variance, will be excluded in the first round of searching, and will be fixed after their estimates are improved by the already fixed ambiguities [36]. From this point of view, this approach is superior to the first one, especially in the case that the first one does not work properly, i.e., cannot fix all ambiguities at once.

We adapt the stepwise fixing approach to the ambiguity resolution of triple-frequency GNSS observations to resolve the integer ambiguities step-by-step according to their wavelength, and to investigate its fixing efficiency.

After applying the ambiguity mapping from the original ambiguity $\left(b_{1},b_{2},b_{3}\right)$ to the EWL, WL and L1 ambiguities $\left(b_{EW},b_{W},b_{1}\right)$, we obtain the normal equation for fixing. In order to facilitate the distinction between the DD-ambiguity parameters and other non-DD-ambiguity parameters, x,y and other non-ambiguity parameters are collectively referred to as X. The normal equation system for ambiguity fixing reads as

(10) $$N_{a}\hat{X}=\left[\begin{array}{cccc} N_{XX} & N_{Xb_{EW}} & N_{Xb_{W}} & N_{Xb_{1}}\\ & N_{b_{EW}b_{EW}} & N_{b_{EW}b_{W}} & N_{b_{EW}b_{1}}\\ & & N_{b_{W}b_{W}} & N_{b_{W}b_{1}}\\ sym & & & N_{b_{1}b_{1}} \end{array}\right]\cdot \left[\begin{array}{c} X\\ b_{EW}\\ b_{W}\\ b_{1} \end{array}\right]=\left[\begin{array}{c} W_{1}\\ W_{2}\\ W_{3}\\ W_{4} \end{array}\right]$$

(11) $${Q^{\prime }}_{NN}=\left[\begin{array}{cccc} Q_{XX} & Q_{Xb_{EW}} & Q_{Xb_{W}} & Q_{Xb_{1}}\\ & Q_{b_{EW}b_{EW}} & Q_{b_{EW}b_{W}} & Q_{b_{EW}b_{1}}\\ & & Q_{b_{W}b_{W}} & Q_{b_{W}b_{1}}\\ sym & & & Q_{b_{1}b_{1}} \end{array}\right],{Q^{\prime }}_{NN}=N_{a}^{-1}$$

The ambiguity searching should be carried out at least three times, i.e., for EWL, WL and B1, respectively, if no partial fixing is required. After each successful fixing, the solution is updated by inserting the fixed integer values into the normal equations and the updated ambiguities and their Q matrix are employed in the next searching.

Taking the first step as example, applying LAMBDA searching approach to $Q_{b_{EW}b_{EW}}$ and $b_{EW}$ of the float solution, we obtain the integer EWL ambiguity vector ${b^{\prime }}_{EW}$. Inserting the integer into the normal equations, the normal equations with WL and B1 ambiguities can be expressed as:

(12) $${N^{\prime }}_{a}{\hat{X}}^{\prime }=\left[\begin{array}{ccc} N_{XX} & N_{Xb_{W}} & N_{Xb_{1}}\\ & N_{b_{W}b_{W}} & N_{b_{W}b_{1}}\\ sym & & N_{b_{1}b_{1}} \end{array}\right]\cdot \left[\begin{array}{c} X\\ b_{W}\\ b_{1} \end{array}\right]=\left[\begin{array}{c} W_{1}-N_{Xb_{EW}}\cdot {b^{\prime }}_{EW}\\ W_{3}-N_{b_{W}b_{EW}}\cdot {b^{\prime }}_{EW}\\ W_{4}-N_{b_{1}b_{EW}}\cdot {b^{\prime }}_{EW} \end{array}\right]$$

Solve the normal equation system, the updated solution with fixed EWL ambiguities is derived. Then we can repeat the above process for WL ambiguities by applying LAMBDA searching to the estimates and their co-variance matrix.

There is also algorithm for updating the solution with fixed ambiguities without resolving the normal equations again, which can be find in a number of references, i.e., in Teunissen [21]. Considering the computer capacity nowadays and for better understanding, we resolve the normal equations in this study.

### 3.3. Ambiguity Resolution Scheme

Based on the above discussion, there could be three ambiguity fixing schemes for triple-frequency data processing: integrated fixing of DD ambiguities of original signal frequencies, integrated fixing of DD ambiguities of combined frequencies with longer wavelength, and stepwise/cascading fixing of DD ambiguities of combined frequencies, referred as to Scheme 1 (S1), Scheme 2 (S2) and Scheme 3 (S3), respectively. Here the combined frequencies are EWL, WL and one original frequency B1.

As uncombined observations are mostly suggested for multi-frequency data processing for making use of ionosphere constraints and to avoid the selection of ionosphere-free combinations for triple-frequency data, S1 is the natural way for ambiguity fixing. However, mapping to EWL, WL and B1 for fixing is believed more efficient because of the longer wavelength of EWL and WL ambiguities. As already mentioned, the LAMBDA method tries to reach the best linear integer combination of ambiguities to reduce the correlation to obtain exactly the confidence ellipsoid containing the best integer candidate. The combination includes also those among the ambiguities of different frequencies. This means that LAMBDA method may automatically achieve the same or similar combination no matter whether original or mapped ambiguities are employed. Therefore, S2 is designed for a numerical clarification of such a misunderstanding. S3 is actually to improve the fixability of ambiguities sequentially by fixing the most easily to be fixed ones in advance. It should be the best strategy if enough satellites are observed.

For all the three fixing schemes, DD observations of the signal frequencies are used and normal equation with SD ambiguities are formed as in Equation (6). Then the SD ambiguities are mapped into DD ambiguities using Equation (8). For Schemes 2 and 3 the DD ambiguities of the signal frequencies are further mapped into EWL, WL and B1 with Equation (10). In this way, the float solution of the three schemes are exactly the same, so that we can evaluate the efficiency of the ambiguity fixing.

## 4. Experimental Validation

In order to validate the implemented algorithm for medium-range RTK positioning, especially the comparison of the three ambiguity resolution schemes, data of baselines with various inter-station distances are collected and processed using the three fixing scheme with dual- and triple-frequency data of GPS and BDS and in static and kinematic mode, respectively. the atmosphere after double difference can effectively eliminate for short baseline, so the short baseline cases get conclusion is almost not affected by the atmospheric. However, atmosphere is the main influence factors for the long baseline. Whether conclusions change as baseline length raising, this is also one of the research objects in this paper. In this paper, the conclusions of the above three schemes were first obtained by statistical analysis of the short baseline data, and then further verified in the long baseline, and the results are analyzed and investigated.

### 4.1. Experimental Data

In total four baselines are selected with from different networks with baseline length of 258 m, 22 km, 47 km and 53 km representing short and middle-range baselines, respectively. Baseline A is the data collected using the receiver, both stations are equipped with SinoGNSS K708. Baseline B between station CASM and LFSL stations are equipped with UNICORE UB4B0 receiver. Baseline A and B are measured data. Baseline C1 between LNSB and LNTL are from the Liaoning CORS network, all with Trimble Net R9. Baseline C2 of Guangdong CORS network data, the receivers are SinoGNSS K708.

All the receivers are capable of tracking GPS and BDS triple-frequency data and the sampling rate is 1 Hz. However, as not all GPS satellites are transmitting L5 signals only dual-frequency data is utilized for GPS in this study. The information of the baselines is listed in Table 1. Although baselines C1 and C2 are of similar length, they are in the north and south of China, respectively, and therefore may have quite different ionosphere impacts.

Among the four baselines, the average number of GPS observables is about 7. Baseline B and C1 have almost the same number of BDS and GPS satellites, while Baseline A and C2 have at least two more BDS satellites on average than GPS.

### 4.2. Data Processing

The data of each baseline is divided into 10 min sessions and processed independently in static and kinematic mode, respectively. For each 10-min session, data of dual-frequency of GPS and both dual- and triple-frequency of BDS are processed with the three ambiguity fixing schemes. The solutions are named according to the systems (BDS, GPS), frequencies (TF, DF) and fixing schemes (S1, S2, S3) accordingly. In total, for each session we have nine solutions for either static or kinematic processing, namely BDS-TF-S1, BDS-TF-S2, BDS-TF-S2; BDS-DF-S1, BDS-DF-S2, BDS-DF-S3; and GPS-DF-S1, GPS-DF-S2 and GPS-DF-S3. For all the solutions, the epoch interval is 1 s and cutoff elevation is 15 degree. In the data preprocessing, outliers are detected according to the posterior phase residuals and residuals larger than 0.5 cycles is considered as cycle slips which will not be repaired but modelled with a new ambiguity parameter. For making the fixing decision, RATIO-test is employed. For the float ambiguity vector b, and ambiguity variance matrix $Q_{bb}$, two integer ambiguity candidates with the smallest quadratic form ${\left(n-b\right)}^{T}Q_{bb}^{-1}(n-b)$ are selected. The proportion of the value of the second smallest quadratic form with respect to the smallest one is defined as RATIO. If RATIO is larger than a threshold, the integer candidate with the smallest quadric form is the fixed integer vector. This threshold is 2.0 in this study. In order to verify the ambiguity resolution performance, RATIO, TFFS and the rate of correct fixings are used as indices for its convergence and fixing reliability, respectively. The correct fixing rate is defined as the percentage of the correctly fixed epochs as:

(13) $$P=\frac{N_{suc}}{N_{all}}$$

where $N_{suc}$ represents the number of epochs where ambiguities are correctly fixed, and $N_{all}$ the total number of epochs. The correctness of a fixing is confirmed by full consistence of the integer ambiguities with that derived from all data of the baseline in static mode using the high-precision data processing software package PANDA [37].

Atmospheric errors have a significant impact on the success of ambiguity resolution, especially in the case of long baselines. The influence of atmospheric errors on the short baseline, for example shorter than 10 km, is negligible due to the strong spatial correlation between the two stations. For middle and long baselines, the troposphere and ionospheric delays are estimated with constraints, the degree of improvement depends on the accuracy of the imposed tropospheric and ionospheric constraints [8]. The tropospheric zenith delay is constrained with an initial STD of 2 mm, and a power density of 10 mm/sqrt(hour) for Baseline B, C1 and C2. For the ionospheric delay parameters, the a priori value for the zenith direction is selected according to the linear function: $\sigma_{I}[\mathrm{cm}]=0.04\cdot length[\mathrm{km}]$ [34,38], then the power density of the temporal ionospheric variation is calculated from ionosphere combinations of phase observations of the previous epochs and a simple mapping function 1/sin(E) is applied to obtain the values for the slant delay parameters of each satellite.

### 4.3. Statistics of Ambiguity Resolution

We first have a close look at the statistics of ambiguity fixing, namely the TFFS and the correct fixing rate for both static and kinematic processing. As the statistics for both processing modes have very similar behavior, we will present them together and more detailed analysis will be carried out for the kinematic mode since we are more interested in medium-range RTK positioning.

Table 2 shows the statistics of TFFS and the correct fixing rate of the nine solutions in both the static and kinematic mode for Baseline A, in which the TFFS are in unit of epochs and it is consistent with the length of time in seconds as the sampling rate is 1 s.

From Table 2, for the short baseline, the ambiguity convergence times of the fixing scheme S1 and S2 are the same for both GPS and BDS, and DF and TF observations. The stepwise fixing scheme S3 can further improve the fixing by reducing the ambiguity convergence time for BDS-DF and GPS-DF data, from 4.0 s to 3.0 s and 5.0 s to 4.0 s, respectively and increasing the correct fixing rates, although the magnitude is rather small. From the statistics of the kinematic positioning shown on the second half of the table, only few more epochs are needed for the first fixing and the correct fixing rate is slight decreased, and the overall behavior of the three processing scheme is almost the same compared with that of the static mode.

The same statistics for the middle range baseline B are listed in Table 3. From Table 3 we also observed the similar phenomena as for Baseline A that the ambiguity convergence of S1, S2 are the same and S3 brings additional improvement compared to S1 and S2 BDS. The triple-frequency data shorten the TFFS very significantly for this middle range baseline which is hardly noticeable for the short baseline. It should be pointed out that the contribution of S3 is obvious, which is rather small in the short baseline. Compared with the static mode, the convergence gets clearly slower and the correct fixing rate decreases of several points in percentage as well for the kinematic mode, except the triple-frequency BDS data, especially the processing scheme S3 where fixing can be achieved again within a few epochs on average as for the short baseline.

Table 4 and Table 5 show the same statistics for the two medium-range baselines: Baseline C1 and C2 where remaining ionospheric and tropospheric delays in differenced observations get larger and must be handled properly as above described. From Table 4 and Table 5 we can again obtain the similar conclusions as that for the medium baseline B that S1 and S2 have the same fixing performance for both GPS and BDS and for all data types DF and TF, and the impact of the third-frequency data is even stronger, whereas S3 can reduce the TFFS for DF data markable and keep the TFFS for TF of few seconds as for the short baseline.

From the overall statistics of ambiguity resolution, S1 and S2 have the same performance in terms of TFFS and the correct fixing rate for all the solutions: DF and TF of BDS data and DF of GPS, while S3 overperforms S1 and S2 obviously except the short baseline. When we have a close look at the statistics of the S3 solutions, the performance seems correlated with the baseline length, the advantage of S3 is better over longer baselines. One possible reason could be that over short baselines the float solution is already very strong and precise, so that sequential fixing could not further improve the solution.

**Figure 1:**
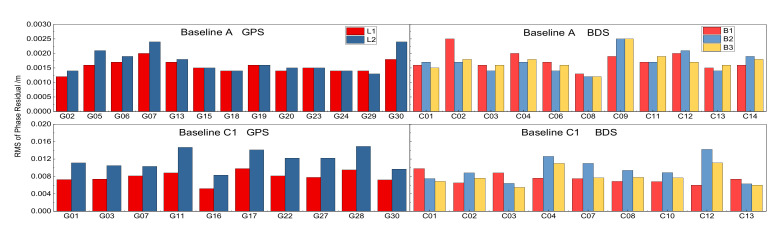
RMS of the posterior phase residuals for each individual satellite and frequencies of the float solutions of the short Baseline A (**top**) and C1 (**bottom**) processed in static mode.

To confirm this possible reason, we furthermore calculated the RMS of posterior phase residuals of the float solutions of the baseline A and C1 and depicted on Figure 1. The RMS of the residuals of the short Baseline A shown in the top panel is only about 0.003 cycles which is consistent with the observation noise as most of the biases are well cancelled by the between-station differencing. The RMS of the two systems are comparable and BDS has a slight larger RMS. For the medium-range Baseline C1 the RMS increases to 0.02 cycles because of inaccurately modeled biases, such as atmospheric delays, multi-path and even satellite orbits. As is well known, larger phase residuals will make the B1 ambiguity fixing difficult, therefore fixing EWL and WL in advance will improve the solutions for B1 fixing. That is why the advantage of the stepwise fixing S3 over S1 and S2 can be better reflected over medium-range baseline.

**Figure 2:**
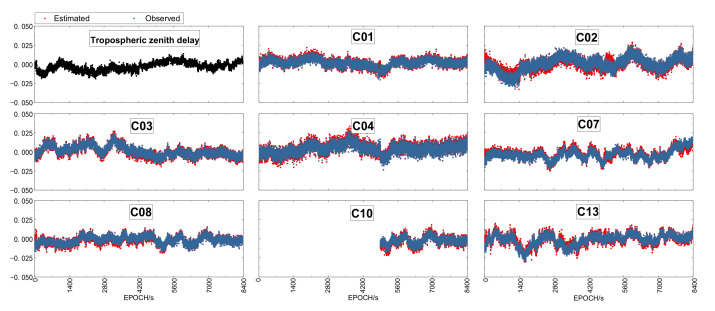
The top-left panel shows the estimated tropospheric zenith delay, and the other panels illustrated the ionospheric delays derived from B1/B2 phase observations aligned to the and the estimated slant ionospheric delay (red) for all the satellites over baseline C1 processed in kinematic mode in 10-min sessions. The estimates before ambiguity-fixing are not plotted.

The atmospheric delay errors could be one of the major factors affecting the ambiguity resolution, especially in the case of long baselines. Taking Baseline C1 processed in kinematic mode and in 10-min sessions as an example, the estimated tropospheric zenith delays (top-left panel) and the slant ionospheric slant delays of all satellites are illustrated in Figure 2. The Ionospheric delays derived from the B1 and B2 phase observations, referred to as “observed” are also shown in the corresponding panels. The estimates before the ambiguity-fixing are not plotted, as there is usually a big jump in the estimated time series due to the big change of ambiguities from their float values to the integer ones. Therefore, there is usually a short gap between two adjacent sessions which is hardly visible due the high sampling rate.

As can be seen from the Figure 2, the remaining tropospheric zenith delay is rather stable and fluctuates within (−0.01 m, 0.01 m) with a slight increasing trend. The estimated ionospheric slant delays are of the similar amplitude, but the temporal changing is much fast. More important is that both the estimated and observed agree with each other very well after aligned by a constant. From this example, the ionospheric and tropospheric delays should be estimated in order to reduce their impact on the other parameters, especially the ambiguity parameters and their fixing.

### 4.4. Comparison of Fixing Index RATIO

**Figure 3:**
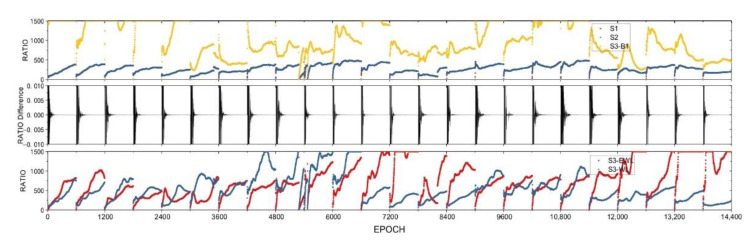
Time series of ambiguity-fixing indicator RATIO of the fixing scheme S1 and S2 (**top**), and their differences (**middle**), and that of EWL and WL fixing of S3 (**bottom**), the RATIO of B1 fixing in S3 is also shown at the top panel for the BDS-TF solutions of Baseline A in static mode.

To further compare the three fixing schemes, it is worth to check the fixing index RATIO at each epoch. Taking the BDS-TF solutions of Baseline A and Baseline C1 as example, the RATIO time series of S1 and S2 and S3 of Baseline A are depicted in Figure 3, where S3 has three RATIOs with respect to the fixing of EWL, WL and L1 ambiguities, in order to express the state of RATIO time series more clearly, we set an upper limit of 1500 for RATIO value. From the top panel of Figure 3, the RATIO time series of S1 and S2 are almost the same, their differences shown in the middle panel are within ±0.01. This clearly proves that the preset integer transformation, i.e., from raw to EWL and/or WL ambiguities, has almost no influence on the ambiguity fixing using LAMBD method. Theoretically, LAMBDA algorithm can find out the best Z transformation to narrow the searching space. However, when we compare the transformation matrix, the mapped ambiguities for S1 and S2 are not exactly the same. The reason is most likely the numerical calculation and round from real-value to integer value of the z-transformation. However, it should be further investigated in future.

In the processing with S3, EWL, WL and B1 are fixed sequentially and the solution is updated after each fixing. The RATIO of EWL and WL fixing of S3 shown in the bottom panel is on average about 2–3 times larger than that of S1 and S2. This reflects that EWL as well as WL can be fixed much more easily than fixing all ambiguities together. After both EWL and WL are fixed, the updated solution with B1 ambiguities is improved, the related ambiguities can be fixed with a larger RATIO overall than that of S1 and S2 which can be seen from the top panel of Figure 3.

**Figure 4:**
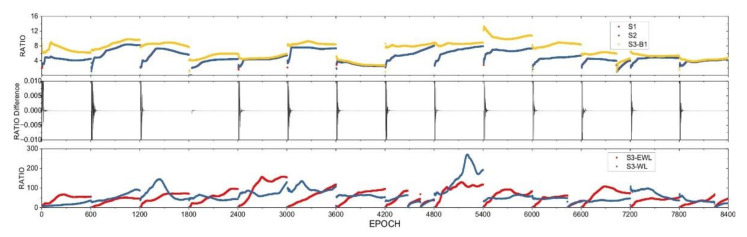
Time series of ambiguity-fixing indicator RATIO of the fixing scheme S1 and S2 (**top**), and their differences (**middle**), and that of EWL and WL fixing of S3 (**bottom**), the RATIO of B1 fixing in S3 is also shown at the top panel for the BDS-TF solutions of Baseline C1 in kinematic mode.

Correspondingly, the RATIO time series of S1 and S2 and S3 are depicted in Figure 4 for the BDS-TF solutions of Baseline C1 in static mode. From Figure 4, the RATIO time series show very similar characteristics except the RATIO values get a little bit smaller due to estimating more parameters in static mode.

The same studying is carried out to the other three baselines and we reveal the same fact. For the above four baselines, S1 and S2 are equivalent in the ambiguity convergence measured by the fixing index RATIO in this experiment, and S3 performs usually better than S1 and S2, regardless of GNSS systems, data types and the positioning modes, especially for medium-range baselines.

In summary, regardless of the length of baselines and the use of DF or TF observations, the ambiguity convergence time indicators of S1 and S2 are the same, and the preset Z transform has almost no effect on the LAMBDA method, while the scheme S3 is superior to S1 and S2 for multi-frequency data.

### 4.5. Positioning Accuracy

The position differences of the fixed solutions with respect to the static solution using all the observations can be used to qualify the positioning accuracy. S3 scheme is superior to S1 and S2 scheme in terms of ambiguity resolution, in order to more clearly and intuitively reflect the positioning result accuracy of multi-frequency data, and in view of the equivalence between S1 and S2 scheme, we use the positioning result of S1 scheme which is superior to the suboptimal performance to calculate the RMS value. The RMS of the position differences of the S1 is calculated for the four baselines using BDS-TF, BDS-DF and GPS-DF observations and listed in Table 6 for static and kinematic processing mode in unit of m.

It can be seen from the table that in static mode the four baselines are better than 2 cm in the north and east directions, and 3 cm in the up direction; and in the kinematic mode similar accuracy can be accomplished in N, E direction and better than 5 cm in up directions.

The positioning results using BDS triple-frequency and dual-frequency data are also compared on Baseline A and B. They are very similar and comparable with GPS dual-frequency. That means the advantage of triple-frequency data in positioning accuracy is hardly visible.

### 4.6. Further Comparison of Ambiguity Resolution Strategies

The core of the LAMBDA method is the reduction process of the correlation of the ambiguity variance covariance matrix through the Z transformation. The final result of Z transformation is the diagonalization of the ambiguity factor matrix. Taking the static processing of BDS triple-frequency positioning of Baseline A as example, the influence of S1, S2 and S3 schemes on Z transformation is discussed for a better understanding of their performance.

**Figure 5:**
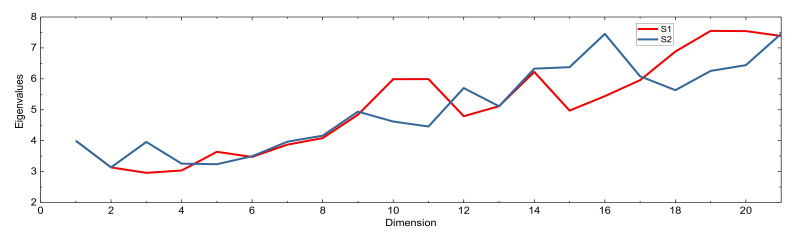
Example of the Eigenvalues of the covariance matrix after Z transformation of S1 and S2 for Baseline A.

Figure 5 is the eigenvalue obtained after reducing the correlation. It can be inferred that S1 and S2 are similar in ellipse flatness from the definition of the condition number after Z transformation in Figure 5. This indicates that the external preset integer transformation applied in S2 has little effect on the correlation reduction of the entire ambiguity search space. However, they are not exactly the same, that means the realization of the best Z-transformation is not unique although theoretically it should be. One possibility could be that the numerical computation from a real-valued to an integer-valued transformation may depends on the starting ambiguity types. This should be further studied in the future. Of course, the final estimated integer ambiguities of original frequencies must be the same for all the three strategies, so that the positioning accuracy of the fixing solutions can be guaranteed.

**Figure 6:**
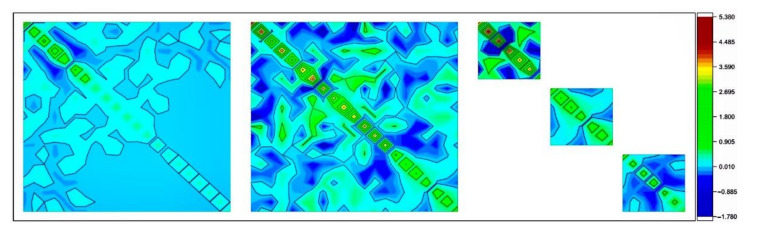
Element values of the covariance matrix after Z-transformation for S1 and S2 and S3 from left to right, respectively. S3 has three blocks for EWL, WL and B1 sequentially.

In Figure 6, from left to right are the two-dimensional diagram of the ambiguity cofactor matrix after Z-transformation of the 33rd epoch obtained from the three schemes: S1, S2 and S3. For S3, there are three blocks with respect to the EWL, WL and B1 ambiguities, as they are fixed in three sequential steps, and the cofactor matrices are based on the updated solution by introducing the integer ambiguities fixed in the previous blocks. The colors represent the values of the corresponding matrix elements and non-diagonal elements illustrate the correlations of the ambiguities. Therefore, after Z-transformation, the smaller the non-diagonal elements, the higher the degree of diagonalization of the matrix, and the better the effect of correlation reduction.

From Figure 6, although the cofactor matrix elements of the EWL ambiguities are larger than that of S1 and S2, the element values of the WL ambiguities and the B1 ambiguities are obviously smaller than those of S1 and S2. This is clearly the contribution of previously fixed integer ambiguities which enhances the solutions and reduces the correlations among the parameters. The theoretical principle can be found in [21] in which it is pointed out that the conditional standard deviations of the optimal combination of ambiguities are very small, and it is feasible to use this advantage to calculate the correct ambiguity within a few epochs. That is why in this study we have designed the S3 scheme to verify this conclusion from the experimental point of view.

**Figure 7:**
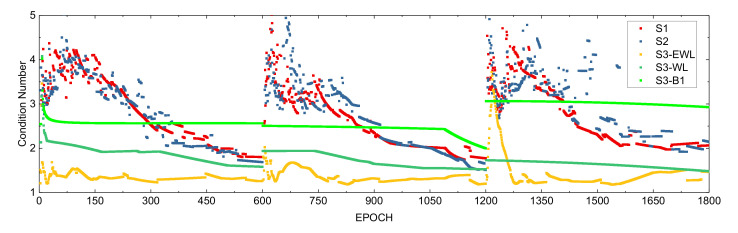
Number of conditions for ambiguity resolution.

The condition number is the ratio of the maximum eigenvalue to the minimum eigenvalue of the normal equation (≥1). The closer it is to 1, the closer the major and minor axes of the ellipsoid are, and the more they tend to be a sphere. Figure 7 is the number of ambiguity resolution conditions for each of S1, S2 and S3. The condition numbers of the S1 and S2 schemes have a relatively small difference, which means that there is little difference between the two in the searching ellipsoid. The condition number obtained by the S3 scheme to solve the ambiguity of the extra-wide lane is the smallest overall. The search ellipse of the wide lane ambiguity search is still smaller than S1 and S2. For the search interval of the original ambiguity, after the first two steps of correction, the accuracy of floating solution of the original ambiguity has been well corrected. Compared with S1 and S2, although the original ambiguity condition number in S3 is larger, it is smaller than S1 and S2 in the initial stage. It can be seen from Figure 3 that in terms of the Ratio value, the ambiguity obtained by the S3 scheme is more reliable, and the experimental results show that this conclusion is valid.

Since ambiguities of long wavelength have the advantage to be fixed and integrated searching of very high dimension of ambiguities could result into computational difficulty, so S3 should be a better choice to effectively shorten the fixed time of multi-frequency ambiguity resolution.

## 5. Conclusions

In the comparative study of the three triple-frequency ambiguity resolution schemes, medium-range RTK is realized by applying adapted temporal constraint to ionospheric slant delay parameters and tropospheric zenith delay parameters. Starting from the same normal equation as the uncombined and undifferenced carrier phase ambiguities, the normal equation with the desired double-differenced ambiguity parameters are generated through ambiguity mapping in order to have a fair comparison. Three fixing schemes, namely S1 fixing all ambiguities of original frequencies simultaneously, S2 fixing all ambiguities of the EWL, WL and one original frequency together, and S3 fixing the same ambiguities as S2 but in a cascading way, are applied to four baselines of 258 m, 22 km, 47 km and 53 km for using BDS triple-frequency and dual-frequency data and GPS dual-frequency data and in both static and kinematic mode. The data is divided into 10 min pieces or sessions and processed independently in order to obtain the TFFS and the correct fixing rate as evaluation indicators.

From the statistics of the ambiguity fixing, S1 and S2 have almost the same fixing performance for all the baselines and sessions and their fixing RATIO time series agree with each other. That means for the integrated searching mapping to ambiguities of longer wavelength does not improve the correct fixing rate, as LAMBDA method searches for the best integer combination for reliable fixing. We also found that the integer transformation matrices from LAMBDA method are not exactly the same for S1 and S2 which should be investigated further.

The cascading scheme S3 overperforms S1 and S2 for all baselines and sessions and its advantage is more significant over longer baselines due to the existence of inaccurately modelled biases and possibly also large noises caused by station environment. The RATIO of S3 of EWL and WL are several ten times larger than that of S1 and S2 and even the RATIO of S3 B1 fixing is also obviously larger than that of S1 and S2 because the float solution of S3 B1 is already improved by the fixed EWL and WL ambiguities.

The efficiency of BDS triple-frequency carrier phase ambiguity resolution is significantly better than that using dual-frequency observation data. For the medium-range baselines in the experiment, the TFFS is always shorter than 10 s on average. This could further extend inter-station distance of medium-range RTK, so that a single reference station could cover a larger area.

In terms of positioning results, the positioning accuracy, measured by the RMS of the position differences of the solutions using dual-frequency data and triple-frequency data is almost the same and the improvement is hardly visible.

## Figures and Tables

**Table 1:** Information of Baselines Employed in the experiment.

Baseline	Length	Start Time	Duration (min)	Receiver	Location Lat. Long. (^o^)
A	258 m	5 October 2020 02:01:00	240	SinoGNSS K708	Liaoning, China 42.0, 121.4
B	22 km	30 March 2019 00:0019	120	UNICORE UB4B0	Beijing, China 39.5, 116.2
C1	47 km	15 November 2017 18:00:00	140	Trimble NetR9	Liaoning, China 42.0, 123.3
C2	53 km	5 June 202020:00:00	220	SinoGNSS K708	Guangdong, China 22.5, 113.5

**Table 2:** Statistics of ambiguity fixing performance of Baseline A, Sampling rate 1 s.

TFFS(s)		BDS-TF			BDS-DF			GPS-DF		
		S1	S2	S3	S1	S2	S3	S1	S2	S3
Static	Median	2.0	2.0	2.0	4.0	4.0	3.0	5.0	5.0	4.0
	MAX	5.0	5.0	2.0	8.0	8.0	6.0	10.0	10.0	9.0
	MIN	2.0	2.0	2.0	2.0	2.0	2.0	2.0	2.0	2.0
	Rate (%)	99.7	99.7	99.8	99.4	99.4	99.6	99.3	99.3	99.4
Kinematic	Median	4.0	4.0	2.0	7.0	7.0	5.0	11.0	11.0	9.0
	MAX	5.0	5.0	4.0	17.0	17.0	12.0	22.0	22.0	18.0
	MIN	2.0	2.0	2.0	2.0	2.0	2.0	3.0	3.0	4.0
	Rate (%)	99.5	99.5	99.8	98.4	98.4	99.3	98.2	98.2	98.4

**Table 3:** Statistics of ambiguity fixing performance of Baseline B, Sampling rate 1 s.

TFFS(s)		BDS-TF			BDS-DF			GPS-DF		
		S1	S2	S3	S1	S2	S3	S1	S2	S3
Static	Median	7.0	7.0	3.0	30.0	30.0	21.5	20.0	20.0	9.0
	MAX	42.0	42.0	9.0	62.0	62.0	30.0	24.0	24.0	20.0
	MIN	4.0	4.0	2.0	28.0	28.0	4.0	3.0	3.0	4.0
	Rate (%)	99.2	99.2	99.7	96.9	96.9	97.0	98.3	98.3	99.3
Kinematic	Median	10.0	10.0	3.0	40.0	40.0	39.0	30.0	30.0	26.5
	MAX	10.0	10.0	10.0	72.0	72.0	40.0	70.0	70.0	30.0
	MIN	5.0	5.0	2.0	40.0	40.0	5.0	4.0	4.0	4.0
	Rate (%)	98.7	98.7	99.4	94.3	94.3	94.9	96.6	96.6	96.7

**Table 4:** Statistics of ambiguity fixing performance of Baseline C1, Sampling rate 1 s.

TFFS(s)		BDS-TF			BDS-DF			GPS-DF		
		S1	S2	S3	S1	S2	S3	S1	S2	S3
Static	Median	8.0	8.0	7.0	121.5	121.5	20.0	31.0	31.0	11.0
	MAX	39.0	39.0	9.0	188.0	188.0	82.0	87.0	87.0	29.0
	MIN	6.0	6.0	3.0	32.0	32.0	6.0	4.0	4.0	5.0
	Rate (%)	99.0	99.0	99.1	93.4	93.4	96.0	96.2	96.2	98.2
Kinematic	Median	10.0	10.0	9.0	173.5	173.5	44.5	53.0	53.0.	17.0
	MAX	71.0	71.0	18.0	280.0	280.0	111.0	264.0	264.0	154.0
	MIN	7.0	7.0	3.0.	105.0	105.0	6.0	8.0	8.0.	6.0
	Rate (%)	98.6	98.6	98.7	89.7	89.7	92.8	92.4	92.4	93.2

**Table 5:** Statistics of ambiguity fixing performance of Baseline C2, Sampling rate 1 s.

TFFS(s)		BDS-TF			BDS-DF			GPS-DF		
		S1	S2	S3	S1	S2	S3	S1	S2	S3
Static	Median	7.0	7.0	3.0	82.0	82.0	12.0	83.0	83.0	11.0
	MAX	14.0	14.0	9.0	276.0	276.0	47.0	184.0	184.0	72.0
	MIN	6.0	6.0	3.0	4.0	4.0	3.0	5.0	5.0	4.0
	Rate (%)	98.9	98.9	99.6	95.5	95.5	96.8	94.7	94.7	97.4
Kinematic	Median	6.0	6.0	3.0	116.0	116.0	16.0	91.0	91.0	23.0
	MAX	30.0	30.0	18.0	297.0	297.0	105.0	186.0	186.0	59.0
	MIN	7.0	7.0	3.0	12.0	12.0	4.0	7.0	7.0	4.0
	Rate (%)	98.7	98.7	99.6	94.9	94.9	95.6	94.6	94.6	95.9

**Table 6:** The RMS of each baseline position in static model, unit is m.

	Baseline	BDS-TF			BDS-DF			GPS-DF		
		N	E	U	N	E	U	N	E	U
Static	A	0.0009	0.0018	0.0032	0.0018	0.0017	0.0035	0.0014	0.0021	0.0031
	B	0.0120	0.0088	0.0236	0.0152	0.0104	0.0254	0.0096	0.0130	0.0229
	C1	0.0133	0.0127	0.0253	0.0158	0.0137	0.0262	0.01477	0.0182	0.02498
	C2	0.0113	0.0126	0.0200	0.0152	0.0147	0.0283	0.0151	0.0147	0.0260
Kinematic	A	0.0014	0.0021	0.0028	0.0023	0.0027	0.0062	0.0027	0.0032	0.0053
	B	0.0155	0.0126	0.0289	0.0163	0.0129	0.0315	0.0102	0.0138	0.0253
	C1	0.0254	0.0256	0.0440	0.0250	0.0287	0.0517	0.0215	0.0217	0.0442
	C2	0.0158	0.0154	0.0400	0.0159	0.0193	0.0448	0.0154	0.0161	0.0510

## Data Availability

The data presented in this study are available on request from the corresponding author. The data are not publicly available due to official permission is required.
